# Isolation of Live Leukocytes from Human Inflammatory Muscles

**DOI:** 10.3390/mps4040075

**Published:** 2021-10-16

**Authors:** Jerome D. Coudert, Emily McLeish, Anuradha Sooda, Nataliya Slater, Kelly Beer, Shereen Paramalingam, Phillipa J. Lamont, Merrilee Needham

**Affiliations:** 1 Centre for Molecular Medicine and Innovative Therapeutics, Murdoch University, Murdoch, WA 6150, Australia; E.McLeish@iiid.murdoch.edu.au (E.M.); a.sooda@iiid.murdoch.edu.au (A.S.); N.Slater@iiid.murdoch.edu.au (N.S.); K.Beer@iiid.murdoch.edu.au (K.B.); merrilee.needham@health.wa.gov.au (M.N.); 2 Perron Institute for Neurological and Translational Science, Nedlands, WA 6009, Australia; 3 School of Medicine, University of Notre Dame, Fremantle, WA 6160, Australia; 4 Department of Rheumatology, Fiona Stanley Hospital, Murdoch, WA 6150, Australia; Shereen.Paramalingam@health.wa.gov.au; 5 Neurogenetic Unit, Royal Perth Hospital, Perth, WA 6000, Australia; Phillipa.Lamont@health.wa.gov.au; 6 Department of Neurology, Fiona Stanley Hospital, Murdoch, WA 6150, Australia

**Keywords:** myopathy, myositis, autoimmunity, immune cells, isolation, enzymatic digestion

## Abstract

In inflammatory myopathies, the self-reactive immune cells involved in muscle aggression have been studied mostly using histological assessment of muscle biopsy sections; this methodology provides the advantage of visualizing and identifying cells within the tissue, but it does not allow further investigation. To gain access to live and isolated cells, many studies utilized blood samples; however, in the absence of biological tools to discriminate the leukocytes associated with the autoimmune process from those that emerged from responses against pathogens, the information observed on circulating immune cells often lacks in specificity, and thus result interpretation may prove difficult. In order to selectively retrieve self-reactive immune cells, we developed a protocol to isolate live leukocytes from human muscle biopsies, which allows for further analysis using a large range of methodologies. The protocol uses enzymatic digestion to release live leukocytes from freshly collected skeletal muscle samples, followed by filtration and separation of the leukocytes from the myocytes by density gradient centrifugation. The isolated cells can be submitted immediately to various analysis strategies to characterize ex vivo the specific cellular and molecular mechanisms responsible for self-directed immune muscle aggression or may be placed in culture for expansion.

## 1. Introduction

Inflammatory myopathies are a group of chronic conditions that affect skeletal muscles; they involve muscle inflammation, weakness and loss of function. The most common types include dermatomyositis (DM), necrotizing autoimmune myopathy (NAM), polymyositis (PM), sporadic inclusion body myositis (IBM) and myositis overlapping with systemic connective tissue disorders [1]. Biologically, most are associated with autoimmune manifestations characterized by inflammatory infiltrates constituted of activated leukocytes. In IBM, these lymphocytes invade non-necrotic muscle fibers. In NAM, the biopsies are usually acellular with respect to inflammatory cells and marked only by myocyte necrosis and regeneration [2].

To date, studies aiming at describing and understanding the autoimmune response in inflammatory myopathies have mostly investigated immune cells in patient blood samples [3,4]. This approach is non-specific as there is currently no tool to discriminate the autoreactive cells within the pool of activated immune cells in blood. Another approach consists in analyzing muscle tissue sections by microscopy. This also provides only limited information, such as where the cells localize within the muscle and the cell types found in the infiltrates. This is because the cell characterization is based on their morphology and/or on the detection of a surface markers by immunofluorescence or by immunochemistry [5].

In IBM, CD8^+^ T cells and macrophages are the predominating subpopulations of leukocytes that have been detected infiltrating the connective tissue surrounding the muscle fibers (endomysium). T cells also invade non-necrotic muscle fibers [6]. T cells are an essential population of the immune system; they are separated into the CD4^+^ and CD8^+^ subsets; each exerting distinct functions in response to the highly restricted recognition of specific antigenic peptides that are presented by HLA class II and class I molecules, respectively. 

In IBM, myocytes display a strongly up-regulated expression of HLA molecules, particularly class I but also class II; this process is believed to mediate the direct antigenic peptide presentation within skeletal muscles and trigger the production of inflammatory cytokines and cytotoxic activity by infiltrating T cells [7]. Macrophages are also found to abundantly accumulate within IBM muscles [8] with other immune subpopulations, such as neutrophils and B cells being detected with lower numbers [9]. Nevertheless, the characterization of auto-aggressive immune cells remains limited and lacks in precision. Most importantly, in histological studies immune cells are fixed within the tissue sample, which severely restricts the type of tests that may be further performed.

To our knowledge, effective protocols to isolate live and unaltered leukocytes from human skeletal muscles are lacking. We found protocols that meet this description in previous mouse studies [10,11,12,13]. We attempted to transpose these protocols to human muscle samples, and experienced multiple issues. The yield of cell recovery was poor due to weak cell release. In humans afflicted by inflammatory myopathies, skeletal muscles often exhibit fatty tissue replacement, which hinders cell release following digestion. In order to circumvent this issue, we attempted to increase the amount of enzyme used and the duration of the digestion. This resulted in low viability, reduced expansion capability and the apparent reduction of surface marker expression, likely due to motif alterations and impaired antibody binding, thereby, preventing cell characterization by flow cytometry, as previously reported in [14]. In addition, the DNA released by the cells damaged during the biopsy collection and processing and trapped the cells within a viscous suspension that could not be efficiently filtered through 70-micron cell strainers.

Laser capture microdissection is another methodology that has been used to selectively isolate individual immune cells from muscle biopsy cryosections [9]. This approach allows further genetic and transcripts expression analyses, however, it has several limitations. First, it requires very specific pieces of equipment and operator training; secondly, the number of cells that can be retrieved is limited; finally, it involves fixation of the tissue in ethanol; as such, the cells can be neither further tested functionally nor expanded.

We developed a protocol allowing the isolation of live leukocytes from fresh human muscle biopsies. Our protocol only uses common in inexpensive laboratory consumables and equipment, does not require long training to perform, can be achieved within a few hours, enables obtaining live leukocytes, and the number of cells that may be retrieved only depends on the level of infiltration within the muscle tissue. As these cells are invading the diseased tissue, it is likely that they are implicated in autoimmune aggression. 

Once isolated, these leukocytes can be submitted to a variety of analysis methodologies, including viability assays, functional assays and characterization of their phenotype and molecule content by multi-parameter flow cytometry or cytometry by time-of-flight (CyTOF) analysis, genomic single-cell analysis etc. This protocol will open new opportunities to fully investigate muscle-invading leukocytes and better understand the pathogenic mechanisms.

## 2. Experimental Design

In order to retrieve live leukocytes, it is essential to obtain freshly excised muscle biopsy. Ideally, tissue processing should start as soon as possible after collection. We recommend placing the muscle biopsy directly into a jar containing no medium and kept on ice during transportation. Muscle tissues smaller than 0.5 × 0.5 × 0.5 cm in size are unlikely to yield sufficient number of leukocytes. In order to achieve optimal digestion, the muscle tissue processed should not exceed 1 cm^3^ when fat adhesions and infiltrations have been resected.

The complete protocol described here from start until the isolated leukocytes have been counted takes 3 to 4 h. If multiple samples were to be available at the same time, we would not recommend that one operator process more than two biopsies simultaneously. This is to avoid (a) excessive delays between sample collection and the start of processing, (b) extended time with enzymes during the digestion step and (c) an over-lengthy isolation process; a swift isolation of the muscle-infiltrating cells will improve their survival rate and fitness at recovery.

**Figure 1:**
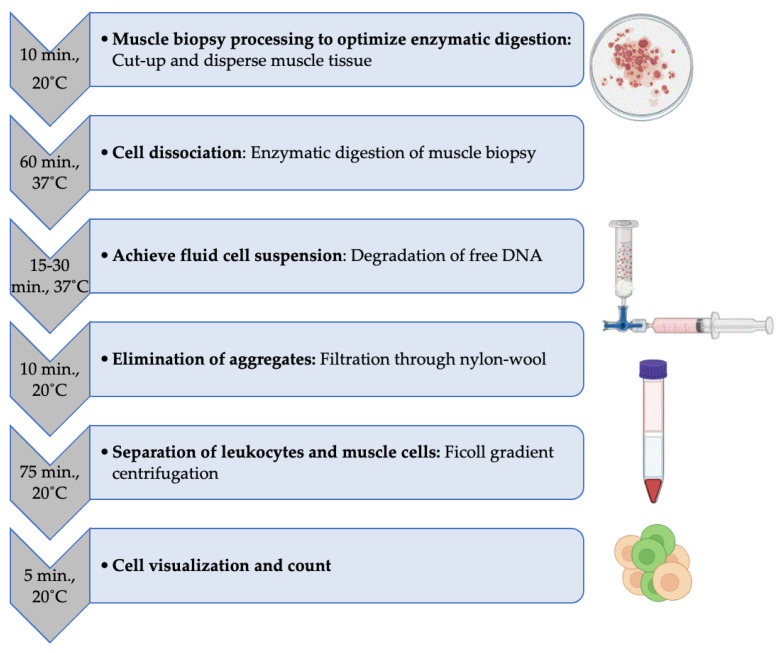
Step-by-step experimental procedure for leukocyte isolation from human muscle biopsy. The average time and temperature condition required for each step are indicated.

The successive steps are as follows (Figure 1):

### 2.1. Materials

**Consumables**

- 10 cm Petri dishes (NUNC, ThermoFisher Scientific, Malaga, WA, Australia, Cat. no.: 150318).
- Scalpel blades (Merck, Bayswater, VIC, Australia, Cat. no.: S2646).
- Pipette tips 1000 μL (ART1000, ThermoFisher Scientific, Malaga, WA, Australia, Cat. no.: MBP2179-05-HR).
- Pipette tips 10 μL (ART10, ThermoFisher Scientific, Malaga, WA, Australia, Cat. no.: MBP2140-05-HR).
- Disposable transfer pipettes (Samco^TM^, ThermoFisher Scientific, Malaga, WA, Australia, Cat. no.: 212-1S).
- Nylon Wool fiber (PolySciences, Gymea, NSW, Australia, Cat. no.: 18369).
- 10 mL Luer-Lock Syringes (Beckton Dickinson, Macquarie Park, NSW, Australia, Cat. no.: 302149).
- Sterilization roll (Steriking^TM^, ThermoFisher Scientific, Malaga, WA, Australia, Cat. no.: R125PK).
- Connecta^TM^ 3-way stopcocks (Beckton Dickinson, Macquarie Park, NSW, Australia, Cat. no.: 394600).
- 15 ml tubes (Greiner, Heidelberg West, VIC, Australia, no.: 188271S).
- 5 mL disposable serological pipettes (Greiner, Heidelberg West, VIC, Australia, Cat. no.: NUN159625N).
- 10 mL disposable pipettes (Greiner, Heidelberg West, VIC, Australia, Cat. no.: NUN170356N).

**Buffer, medium and reagents**

- PBS, pH 7.4, 500 mL bottle (Invitrogen; Malaga, WA, Australia, Cat. no.: 10010-049).
- RPMI 1640, 500 mL bottle (Invitrogen; Malaga, WA, Australia, Cat. no.: 11875093).
- Collagenase P (Roche Diagnostics Australia, North Ryde, NSW, Australia, Cat. no.: 11213865001).
- DNase I (Roche Diagnostics Australia, North Ryde, NSW, Australia, Cat. no.: 10104159001).
- Fetal Calf Serum = FCS (Fisher Biotec Australia, Wembley, WA, Australia, Cat. no.: S-FBS-AU-015).
- Ficoll-Paque Plus (Bio-Strategy, Campbellfield, VIC, Australia, Cat. no.: GEHE17-1440-03).
- Trypan Blue Solution 0.4% (Gibco, Malaga, WA, Australia, Cat. no.: 15250061).

### 2.2. Equipment

No specific brands are required for the equipment listed below:

- P1000 Pipette.
- P10 Pipette.
- Electronic pipette controller.
- Tube Rotator MACSmix^TM^, (Miltenyi Biotec, Macquarie Park, NSW, Australia) *or other model with similar specifications.*
- Incubator set at 37 °C, 5% CO2.
- Centrifuge with swinging-bucket rotor.
- Microscope.
- Neubauer Hematocytometer and coverslip.

## 3. Procedure

Note: Work under a Biosafety cabinet to ensure a sterile working environment for the samples and to keep the operator protected from potentially infectious samples.

### 3.1. Dissection of the Muscle Biopsy

1. Place the muscle biopsy in a 10 cm Petri dish (Video S1).
2. If necessary, separate the adipose tissue that may be attached to the muscle tissue using a sterile scalpel blade, while holding the biopsy in place using sterile dissecting forceps.

Note: Instead, a sterile 1 mL tip mounted on a P1000 pipette may be used to hold the biopsy in place if forceps are not available.

3. Discard the resected adipose tissue fraction.
4. Cut the muscle tissue into small pieces and disperse using the scalpel blade while holding in place with the dissecting forceps.
5. Using a sterile disposable transfer pipette, add 2 mL of sterile PBS onto the muscle fragments, resuspend and transfer into a 15 mL tube.
6. Add another 2 mL of PBS in the Petri dish to rinse and optimize sample recovery; then, resuspend and transfer into the same 15 mL tube.

### 3.2. Enzymatic Digestion of the Muscle Tissue

1. Add 1 mL of Collagenase P (stock solution concentration: 6 mg/mL) onto the 4 mL of muscle tissue suspension in the 15 mL tube, thereby, obtaining a final concentration of 1.2 mg/mL.

Note: Double the volumes (8 mL tissue suspension in PBS + 2 mL Collagenase P) if the biopsy is bigger than 1 cm^3^.

2. Place the tube on a tube rotator. Place another tube of similar weight on the rotator for balancing purpose.
3. Place the rotator in a 37 °C incubator and set to rotate at 10–15 revolutions/minute for 60 min.
4. Resuspend the suspension using a disposable transfer pipette to improve tissue dissociation.

Note: At this stage, the cell suspension has a viscous appearance due to the release of DNA, which needs to be digested to allow the separation of individual cells.

### 3.3. Digestion of the DNA Released in the Medium

1. Take the tube off the rotator.
2. Under the safety cabinet, add 50 μL of DNAse I (stock solution 20 mg/mL; 50 μL = 1 mg; final concentration 0.2 mg/mL).
3. Place back on the rotator and leave to rotate at 37 °C for an additional 15 min.
4. If the buffer remains viscous, add another 50 μL of DNAse I and place back to rotate for another 15 min, otherwise, proceed to the next step.
5. Use a disposable transfer pipette to homogenize the cell suspension by aspirating up and down several times in the 15 mL tube.
6. Using a 5 mL disposable serological pipette mounted on an electronic pipette controller, top up to 10 mL with PBS 2% FCS.

### 3.4. Filtration of the Individualized Cells from the Remaining Tissue Aggregates

1. Screw-in a sterile nylon wool-packed 10 mL syringe to the top connection of a 3-way stopcock (Video S2).
2. Screw-in a 10 mL Luer-Lock syringe to the bottom connection of tap; check that the valve is set correctly and that the flow is only possible between the two syringes.

Transfer the cell suspension into the nylon wool-packed top syringe using a disposable transfer pipette.

4. Gently pull the plunger of the syringe connected to the bottom connection of the 3-way stopcock to aspirate and filter the cell suspension through the nylon wool into this bottom syringe.
5. Unscrew the bottom syringe and gently flush its content (filtered cell suspension) into a new 15 mL tube.
6. Using a 5 mL disposable serological pipette, top up to 15 mL using PBS 2% FCS.
7. Place the tube in a centrifuge, balance the rotor accordingly and spin at 300× *g* for 7 min.
8. Aspirate the supernatant using a 10 mL disposable serological pipette and discard.

Note: Be careful not to disrupt the pellet while doing so; if this occurs, flush back the pipette content into the tube and repeat from step 7.

9. Resuspend the cell pellet in 15 mL PBS to wash off the FCS remnant prior to Ficoll separation.

Note: This step is essential to ensure proper density-based Ficoll gradient separation.

10. Again, place the tube in a centrifuge and spin at 300 × *g* for 7 min.
11. Aspirate the supernatant using a 10 mL disposable serological pipette and discard.
12. Using a 5 mL disposable serological pipette, resuspend the cell pellet in 3 mL a phenol red tainted medium (e.g., RPMI or DMEM).

Note: Although this step may also be performed using PBS, resuspending the cells in a red tainted medium will allow better visualization for next steps.

### 3.5. Separation of Leukocytes from Muscle Fiber Cells

Note: Ficoll separation is based on the difference of density between the cell types that will settle at different levels upon centrifugation; this process is affected by the temperature. It is critical that all the Ficoll and medium used are allowed to set at room temperature. Similarly, the centrifuge must be set at room temperature.

1. Using a 5 mL disposable pipette mounted on an electronic pipette controller, place 3 mL of Ficoll into a new 15 mL tube (Video S3).
2. Using a 5 mL disposable pipette mounted on an electronic pipette controller set on low-speed, carefully overlay the 3 mL of cell suspension onto the Ficoll (3 mL) in order to obtain two clearly defined phases.
3. Place the tube in a centrifuge and spin at 800 × *g* for 15 min with the brake turned off.

Note: Turning the brake off is critical to prevent the leukocyte layer that forms during this centrifugation step to be disrupted by a sudden deceleration. Depending on the model of centrifuge, the rotor will take 20–30 min to come to a stop.

It is also critical that the rotor is perfectly balanced to avoid vibrations that would otherwise disturb the cell layers. The leukocytes constitute the layer situated at the interphase between the red medium and the Ficoll. The myoblasts, erythrocytes, granulocytes and cell debris form the pellet.

4. Aspirate the leukocyte layer using a disposable transfer pipette and transfer into new a 15 mL tube.
5. Using a 10 mL disposable pipette mounted on an electronic pipette controller, top up to 15 mL using PBS 2% FCS.
6. Place the tube in a centrifuge and spin at 300 × *g* for 7 min.
7. Using a 10 mL disposable pipette mounted on an electronic pipette controller aspirate the supernatant and discard.
8. Using a P1000 pipette, gently resuspend the cell pellet by aspirating up and down in 1 mL PBS 2% FCS if the pellet is barely visible.

Note: This volume is adequate for a small pellet, it may be further increased if the cell concentration is too high for counting.

### 3.6. Cell Count of the Isolated Leukocytes

1. Using a P10 pipette, pipet 10 μL of the leukocyte suspension and mix with 10 μL trypan blue.
2. Place 10 μL of the cell/trypan blue mix onto a Neubauer hematocytometer and view under a microscope using the 10X objective lens for counting.

## 4. Expected Results

### 4.1. Number of Immune Cells Recovered

**Figure 2:**
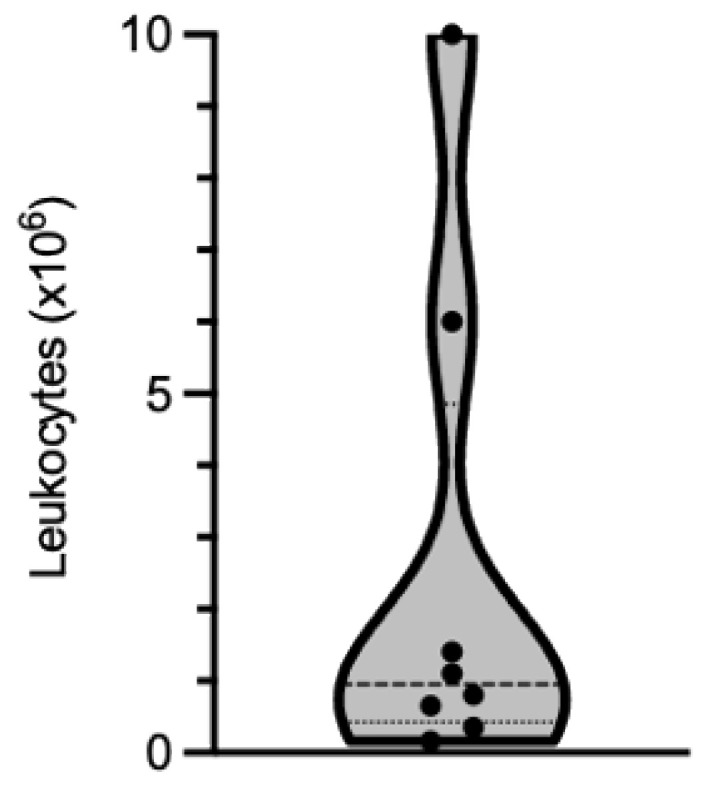
Violin representation of the number of leukocytes isolated from eight muscle biopsies processed independently. Dots show individual values, bold dotted-line the median and the lower and upper fine dotted-lines the 25th and 75th percentiles, respectively.

The yield recovered depends more on the extent of immune cell infiltration in the muscle tissue than on the biopsy size. Based on our experience, the number of cells that can be isolated is highly variable from one biopsy to another. Out of the biopsies that we have processed, the number of leukocytes recovered ranged from 0.165 × 10^6^ to 10 × 10^6^, with a median value of 0.95 × 10^6^ leukocytes (25% percentile: 0.425 × 10^6^; 75% percentile: 4.85 × 10^6^ leukocytes; Figure 2 and Table 1).

### 4.2. Phenotypic Characterization of the Isolated Cells by Flow Cytometry

The composition of the immune cell subpopulations within the recovered leukocytes can be determined by flow cytometry. This procedure is not a part of this protocol but rather may be used as quality control and/or as the initial analysis step of the muscle-isolated leukocytes.

In order to measure accurately the proportion of the subpopulations obtained, 25,000 of the recovered cells may be used. When low cell counts are extracted, as low as 5,000 cells suffice to perform this analysis although the proportions measured will be less accurate.

Note: When performing cell staining with low cell numbers, it is preferably to use a U bottom 96 well plate rather than 5 mL FACs tubes as this will minimize cell loss during the washing step.

- Transfer 25,000 cells (or less) into a well of a U bottom 96 well-plate.
- Stain the cells by adding 50 μL of PBS 2% FCS containing the following antibodies: CD3ε BV510 (UCHT1, Becton Dickinson, Macquarie Park, NSW, Australia), CD4 FITC (OKT4, BioLegend, Wangara, WA, Australia), CD8 APC-H7 (SK1, Becton Dickinson), CD19 APC-FIRE750 (HIB19, Becton Dickinson) and leave during 30 min between 4–20 °C protected from direct light.
- At the end of the incubation period, top up to 250 μL using PBS 2% FCS to wash-off the unbound antibodies. Place the plate in a centrifuge and spin for 5 min at 300× *g*.
- Discard the supernatant without disturbing the cell pellet, which might not be visible.
- Resuspend the cell pellet in 300 μL of PBS 2% FCS and transferred into a FACs tube for analysis on a Beckman Coulter (Lane Cove West, NSW, Australia) Gallios^®^ Flow cytometer using the Kaluza^®^ acquisition software.

**Figure 3:**
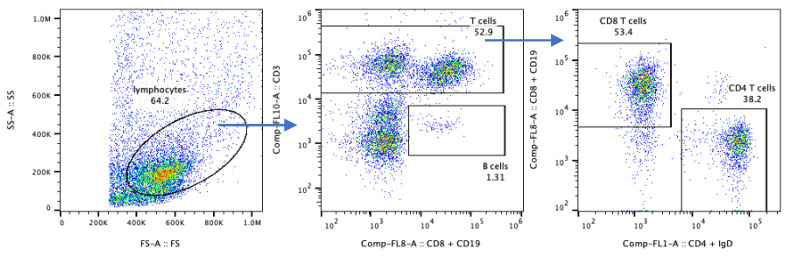
Gating strategy for muscle-isolated immune cell characterization by flow cytometry.

Our flow cytometry raw data were analyzed using the analysis software FlowJo v10.7.1 (Beckson Dickinson, Ashland, OR, USA). The “lymphocyte gate” was defined based on the FS-A/SS-A characteristics (Figure 3, left panel). In this biopsy, we found that 52.9% of the lymphocyte gate cells were CD3+ T cells (middle panel); within which 53.4% were CD4+ and 38.2% CD8+ (right panel), and 1.3% of these lymphocytes were CD19+ B cells (middle panel).

Note: The frequencies measured here are shown only to demonstrate the cell types that can be recovered, as the size of these cell populations is highly variable between biopsy samples.

The surface markers indicated above are not altered by the enzymatic digestion. However, some other epitopes that are targeted by the antibodies used for flow cytometry analysis are more labile and prone to be altered by the enzymes. As such, when a comprehensive staining for flow cytometry analysis is planned, the extracted cells may need to recover for several hours in culture medium to regain an optimal expression profile of unaltered new surface proteins.

## 5. Reagents Setup

### 5.1. Nylon Wool-Packed Syringes

1. Weigh 400 mg of nylon wool fiber on a precision scale.
2. Take a 10 mL Luer-Lock syringe; remove and discard the plunger.
3. Gently pull the fiber until obtaining uniform thin strands.
4. Insert the nylon wool into the syringe and loosely pack to approximately 4 mL graduation
5. Seal inside a sterilization pouch
6. Autoclave at 121 °C for 30 min under at least 15 psi of saturated steam pressure.

### 5.2. Fetal Bovine Serum Inactivation

Serum must be heat-inactivated by placing into a 56 °C heated water bath for 30 min before use.

## Figures and Tables

**Table 1:** Condition and year of diagnosis of the patients who donated a muscle biopsy for this study. The numbers of leukocytes isolated using this protocol are indicated.

Diagnosis	Year of Diagnosis	Leukocyte Isolated (×10^6^)
IBM	2017	1.1
IBM	2008	10
IBM	2017	6
IBM	2020	0.65
IBM	2017	0.8
IBM	2020	1.4
histopathology inconclusive, weak inflammation	2018	0.165
histopathology inconclusive, weak inflammation	2020	0.35

## Supplementary Materials

The following are available online at https://www.mdpi.com/article/10.3390/mps4040075/s1. Video S1: Dissection; Video: S2, Filtration, Video S3, Ficoll.
